# Anti-Inflammatory and Antioxidant Effects of Kelong-Capsule on Testosterone-Induced Benign Prostatic Hyperplasia in Rats

**DOI:** 10.1155/2018/5290514

**Published:** 2018-06-26

**Authors:** Ling Zhang, Xin-Rong Fan, Hui Xie, Qing-Hu He, Yu-Song Nie, Min Zhang, Miao Yan

**Affiliations:** ^1^Department of Pharmacy, The Second Xiangya Hospital, Central South University, Changsha, Hunan Province 410011, China; ^2^Institute of Basic Theory for Chinese Medicine, China Academy of Chinese Medical Sciences, Beijing 100700, China; ^3^College of Integrated Traditional Chinese and Western Medicine, Hunan University of Chinese Medicine, Changsha, Hunan Province 410208, China; ^4^First Clinical Medical College, Shanxi University of Chinese Medicine, Xianyang, Shanxi Province 712000, China

## Abstract

Benign prostatic hyperplasia (BPH) is a common disease in the current ageing male population. This research aims to study the effects of Kelong-Capsules (KLC) on testosterone-induced BPH. Thirty rats were randomly divided into normal group, model group, and three treatment groups. Three treatment groups were given KLC (3.6 g/kg), KLC (7.2 g/kg), and finasteride (0.9 mg/kg), respectively, for 28 days after establishing the animal model. The BPH rat models were evaluated by Traditional Chinese Medicine (TCM) symptoms and prostate index (PI). Results indicated that three treatment groups all alleviated the pathological changes of prostate and kidney at different levels. Compared with the model group, the PI of the groups treated with KLC (7.2 g/kg) and finasteride decreased significantly. The expressions of NF-E2 related factor 2 (Nrf-2) and quinine oxidoreductase (NQO1) in the group treated with KLC (3.6 g/kg) increased markedly (*p* < 0.01). The cyclooxygenase-2 (COX-2) protein expression of the group treated with KLC (7.2 g/kg) was increased (*p* < 0.01). In conclusion, KLC could obviously inhibit the growth of prostate, and KLC (3.6 g/kg) could promote the expressions of Nrf2 and NQO1.

## 1. Introduction

Benign prostatic hyperplasia (BPH) is an age-related disease characterized by proliferation of the epithelial and stroma cell in the prostate gland [1]. It is also the most common cause of the lower urinary tract symptoms (LUTS) [2]. Men with severely enlarged prostate suffer from obstructive and irritative symptoms including nocturia, irreversible bladder failure, and greater risk of acute urinary retention, which affect the quality of life [3, 4]. Although the pathogenesis of BPH has not been fully elucidated, aetiology is explained in ways of ageing, hormonal alterations, growth factors, and inflammatory cell infiltration [5].

The cyclooxygenase-2 (COX-2) enzyme converts arachidonic acid into prostaglandins (PGs), which plays a main role in inflammatory responses [6]. The coexistence of hyperplastic nodules and chronic inflammation in BPH tissue indicates the relationship between BPH and inflammation [7]. Meanwhile, oxidative stress (OS) is one of the mechanisms that trigger the chain of reactions involved in the development of BPH [8]. Many studies have confirmed that reactive oxygen species (ROS), quinine oxidoreductase (NQO1), and other oxidative factors are associated with BPH [9, 10]. NF-E2 related factor 2 (Nrf2) is the key gene that regulates the expression of numerous antioxidant proteins. The COX-2/Nrf2 pathway is one of the most important signal transduction pathways of anti-inflammation and antioxidation.

The therapeutic methods of BPH include drug therapy (alpha-1 blockers, 5-alpha-reductase inhibitors) and operations (prostatectomy, transurethral resection, etc.) [6]. Although many patients take medicine for many years, the progress of BPH could not be halted and eventually develops into complications or the stage requiring surgical intervention [11]. Recently, herbal therapy has become increasingly popular for the good curative and fewer adverse reactions. In our study, Huangqi (Astragali Radix) and Guizhi (Cinnamomi Ramulus) in Kelong-Capsules (KLC) displayed anti-inflammatory effects [12, 13]. Huangqi (Astragali Radix) and soybean isoflavone significantly increased the expression of Nrf2 and the activities of antioxidants [14, 15].

BPH animal model is necessary for in vivo efficacy study. But spontaneous BPH is rare in other species than man. Hormone-induced BPH rat model is proposed, which may be more relevant to the disease [16]. In the previous studies, we successfully established kidney deficiency and blood stasis (KDBS) rat models according to Traditional Chinese Medicine (TCM) Syndrome of BPH [17]. The clinical research demonstrated that KLC could effectively improve the TCM symptom score and the prostate index (PI) [18]. Therefore, we hypothesized that KLC could trigger anti-inflammatory and antioxidant mechanisms via the COX-2/Nrf2 pathway. In this study, we evaluated the efficacy of KLC on testosterone-induced BPH rats and the expressions of anti-inflammatory and antioxidant factors.

## 2. Materials and Methods

### 2.1. Materials

All the herbs were purchased from the First Affiliated Hospital of Hunan Medical University and extracted according to the standard methods recommended by the Chinese Pharmacopoeia (2010). KLC includes 8 kinds of herbs as follows: Dihuang (Rehmanniae Radix Praeparata), Shanzhuyu (Cornus Fructus), Fuling (Poria), Mudanpi (Moutan Cortex), Guizhi (Cinnamomi Ramulus), Fuzi (Aconm Lateralis Radix Praeparaia), Huangqi (Astragali Radix), and Cishi (Magnetitum). The 8 herbs were immersed in 6000 ml of hot water and regathered every 2 hours for 3 times. The solution was concentrated to a small volume at 60°C and then evaporated to dry the extract in a 70°C vacuum drying box. Lastly, the extract powder (200 g) was obtained by smashing the dry extract. The KLC samples (280 g) were composed of extract powder and soybean isoflavone and pumpkin seed oil. The KLC samples were dissolved by 0.5% sodium carboxymethyl cellulose (CMC-Na).

### 2.2. Animals

Adult male Sprague Dawley rats (*n* = 30) weighing 290–320 g were obtained from the Hunan SJA Laboratory Animal Co., Ltd. (specific-pathogen-free grade, certification no. SYXK2014-0012). The rats were housed under constant temperature (23 ± 1°C) and dark-light cycle (12 h : 12 h). All the animal experiments were approved by the Animal Care and Use Committee of the Hunan Academy of TCM and performed according to the Guidelines. Before the formal study, the rats had already been adapted to the laboratory environment for 5 days.

### 2.3. BPH Model and Drug Administration

To exclude the influence of intrinsic testosterone, rats were castrated by removing the testes. Then, penicillin was injected once a day for 3 days to prevent infection. The rat model of KDBS was generated by castration and placement into ice water (7–10°C) for 30 min for 28 days after wound healing. Meanwhile, the BPH rat model was induced by subcutaneous injection of 5 mg/kg testosterone propionate (TP, Tianjin Jinyao Amino Acid Co. Ltd.; batch no. 1301141) for 28 days.

The animals were randomly divided into five groups (*n* = 6, per group): the normal group, the model group, two KLC treatment groups, and the positive control group. Apart from the normal group, the castration had been performed and KDBS models were established in the model and three treatment groups. The five groups were treated with different drugs after the models were established: The normal and model groups received 0.5% sodium carboxymethyl cellulose (CMC-Na). The two KLC treated groups received KLC (3.6 or 7.2 g/kg). The positive control group received 0.9 mg/kg finasteride as 5*α*-reductase inhibitor (Hangzhou Merck Sharp & Dohme Pharmacy Co. Ltd.; batch no. 048743). All drugs were administered to animals via gavage once a day for 28 days. The body weight was measured once a week. At the end of the experiment, the size of the prostatic tissue was recorded. The PI of each rat was the ratio of prostate weight to body weight (mg/g).

### 2.4. Histopathological Analysis

To evaluate morphological changes in the prostate and kidney, tissues were embedded in paraffin, cut into sections of 4 *μ*m thickness, and stained with haematoxylin and eosin (H&E). Morphological changes were observed with light microscopy (Nikon, Tokyo, Japan).

### 2.5. Western Blots

The total protein of the prostate tissue was extracted and concentration was determined by BCA Protein Assay Kit (P001B, Auragene, China). The protein extracts were separated on 10% sodium dodecyl sulfate-polyacrylamide gel electrophoresis (SDS-PAGE) and transferred to polyvinylidene difluoride membranes (PVDF). The membranes were incubated overnight at 4°C with primary antibodies for Nrf2 (1 : 500 dilution; YT3189, Immunoway, USA), NQO1 (1 : 1000 dilution; 11451-1-AP, Proteintech, China), COX-2 (1 : 1000 dilution; 12375-1-AP, Proteintech, China), or *β*-actin (1 : 2300 dilution; LCA01; Auragene, China) after blocking with TBST containing 5% nonfat milk at room temperature for 1 hour. Then, the membranes were washed three times with TBST, incubated with secondary antibodies for goat anti-mouse (1 : 15000 dilution; SA001; Auragene, China) at room temperature for 40 mins, and washed in TBST for 5 times for 3 mins. The bands were visualized with an enhanced chemiluminescence detection kit (P001 WB-1, Auragene, China).

### 2.6. Statistical Analysis

The results were presented with the mean ± standard deviation (SD). Statistically significant differences were determined using a one-way analysis of variance (ANOVA). *p* < 0.05 was considered to indicate a statistically significant difference. These results were analysed with SPSS 17.0 statistical software (SPSS Inc., Chicago, IL, USA).

## 3. Results

### 3.1. Body Weight Variation

**Figure 1:**
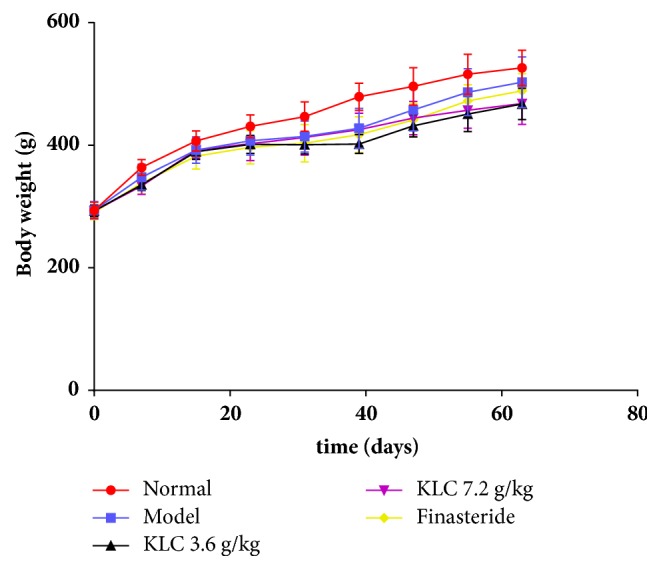
*Body weight changes of rats in the normal, model, and three treated groups*. Data was expressed as the means ± SD (each group, *n* = 6).

During the experiment, rats gained weight significantly. The body weight of the normal group steadily increased until the 62nd day and reached 526.25 ± 28.90 g. The body weight of the model group increased from the initial to the 14th day and reached 392.02 ± 21.60 g. Moreover, the trend of the KLC treatment group at 3.6 g/kg, the KLC treatment group at 7.2 g/kg, and the finasteride group were almost consistent with the model group and reached 389.56 ± 12.79 g, 392.12 ± 12.92 g, and 382.17 ± 21.07 g, respectively. Finally, the body weight of the model increased steadily until the 62nd day to 502.9 ± 41.22 g. The KLC treatment group at 3.6 g/kg, the KLC treatment group at 7.2 g/kg, and the finasteride group slowly gained weight to 467.32 ± 25.53 g, 467.98 ± 33.99 g, and 488.73 ± 27.95 g, respectively (Figure 1).

### 3.2. Model Evaluation

**Figure 2:**
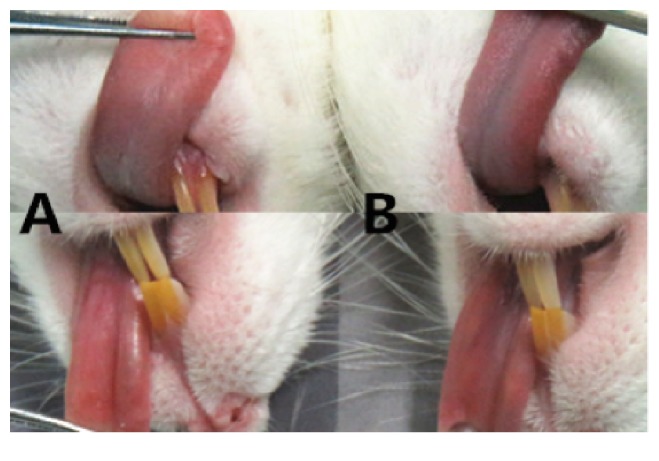
*The changes of tongue colour induced by testosterone propionate after ice bath and castration in rats*. Notes: (A) the normal group and (B) the model group.

Tongue colour was an important evaluative indicator in Traditional Chinese Medicine (TCM) Syndrome Differentiation. The model group showed deeper red than the normal group, as well as looser stools, slower movement, and stagnated weight, or even reduced weight during the modelling period [17]. These clinical symptoms suggested that kidney deficiency and blood stasis model had been established for further studies (Figure 2).

### 3.3. Prostate Size and Prostate Index (PI)

**Figure 3:**
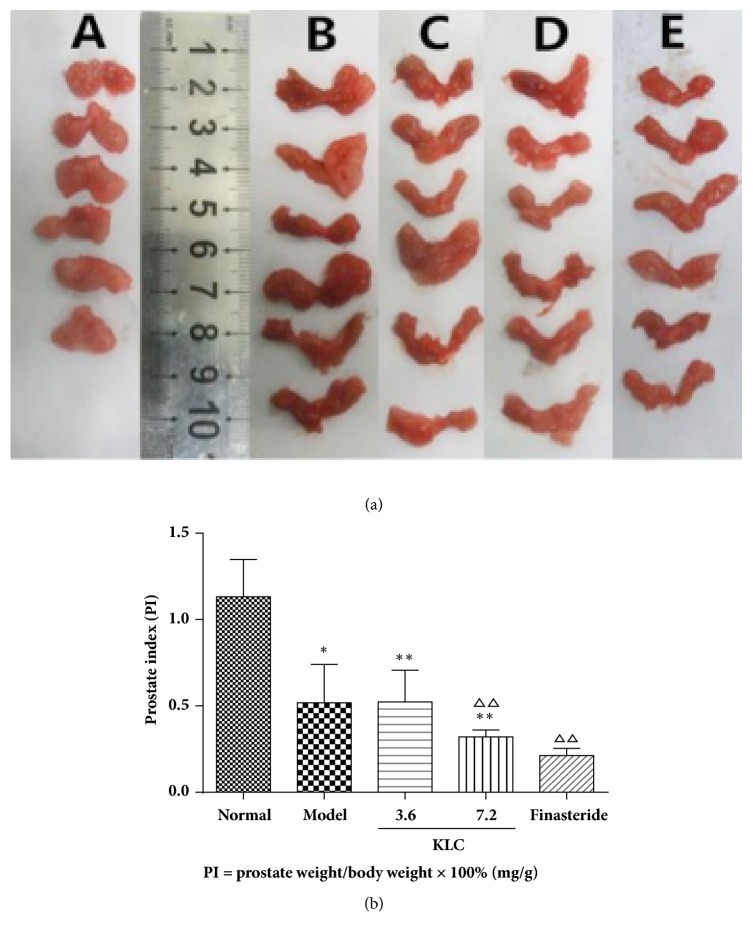
*Effects of KLC on the prostate index (PI) and morphological changes*. Notes: (a) (A) the normal group; (B) the model group; (C) the KLC (3.6 g/kg) group; (D) the KLC (7.2 g/kg) group; (E) the finasteride group. (b) Values were expressed as the means ± SD (each group, *n* = 6); ^*∗∗*^*p* < 0.01 versus normal group; ^△△^*p* < 0.01 versus model group.

The morphological changes of the prostate in the five groups were compared after 62 days. As shown in Figure 3(a), compared with the normal group, there were various degrees of narrowing in the other groups. Obviously, irregular morphological changes of the prostate were observed in the model group. The KLC (7.2 g/kg) and finasteride groups revealed more severe atrophy than the model group. The normal group exhibited an obvious increase of PI compared with the model group and KLC (3.6 and 7.2 g/kg) group (*p* < 0.01). However, there was no significant difference between the model group and the KLC (3.6 g/kg) group (*p* > 0.05). Treatment with KLC (7.2 g/kg) and finasteride had significantly lower PI than the model group (*p* < 0.01; Figure 3(b)). These results showed that castration could inhibit the proliferation of the prostate gland and KLC (7.2 g/kg) and finasteride also had inhibitory effects on the prostate.

### 3.4. Histomorphology of the Prostate and Kidney Tissue

**Figure 4:**
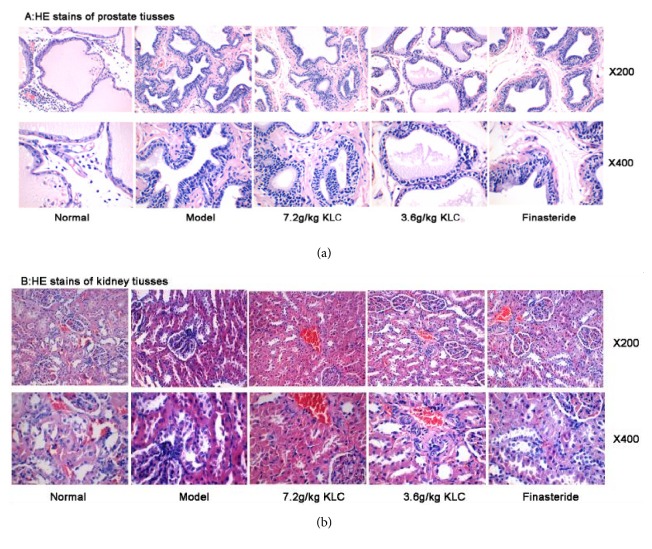
*Effects of KLC on histopathology in BPH rats (HE ×200 and ×400)*. Notes: (a) HE stains of prostate tissues in each group; (b) HE stains of kidney tissues in each group.

In the normal group, there were few inflammatory cells that infiltrated into the prostatic stroma (Figure 4(a)). In the model group, the epithelium layer thickened obviously. There was deformation in the glandular cavities, which was followed by infiltration of some inflammatory cells. The finasteride group exhibited decrease of the epithelium thickness. Compared with the model group, the glandular cavities of the KLC (3.6 and 7.2 g/kg) trended towards the normal group and there were varying degrees of relief on prostatic epithelium hyperplasia. Compared with the normal glomerulus and renal tubules histoarchitecture in the normal group, the kidney of the model group showed tubular necrosis, local oedema, and little inflammatory infiltration (Figure 4(b)).

### 3.5. The Protein Expression of COX-2, Nrf2, and NQO1

**Figure 5:**
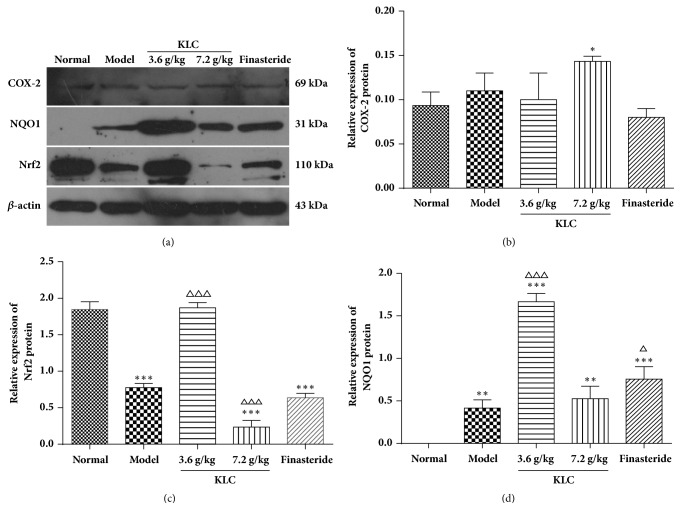
*Effects of KLC on the protein expressions of COX2, NOQ1, and Nrf2 in the prostatic tissue of rats*. Notes: *β*-actin was used as the control group. Data was expressed as the means ± SD (each group, *n* = 3). ^*∗*^*p* < 0.05, ^*∗∗*^*p* < 0.01, and ^*∗∗∗*^*p* < 0.001 versus normal group; ^△^*p* < 0.05, ^△△^*p* < 0.01, and ^△△△^*p* < 0.001 versus model group.

Compared with the normal group, the protein content of COX-2 was significantly upregulated after treatment with KLC (7.2 g/kg; *p* < 0.001). There was no difference in the levels of COX-2 between the model and treated groups (*p* > 0.05; Figure 5(b)). The protein levels of Nfr2 in the model, KLC (7.2 g/kg), and finasteride groups were decreased compared with the normal group (*p* < 0.001; Figure 5(c)). KLC (3.6 g/kg) treatment upregulated the expression of Nrf2 compared with the model group. In contrast, KLC (7.2 g/kg) treatment inhibited Nrf2 expression (*p* < 0.001; Figure 5(c)). The protein levels of NQO1 in the KLC (3.6 g/kg) and finasteride groups were also increased (*p* < 0.01 or *p* < 0.001; Figure 5(d)).

## 4. Discussion

At present, the mechanisms of drug effect on BPH include acting on the diastolic smooth muscle, removing urinary tract obstructions, and reducing the volume of the prostate gland to relieve the symptoms of urinary frequency and dysuria. However, long-term application of chemical drugs was restricted due to the side effects. According to the Chinese medicine theory, kidney deficiency is an essential factor in the formation of BPH, and blood stasis is the pathological basis [19]. In Traditional Chinese Medicine (TCM), tongue diagnosis was one of the most common and valuable diagnostic methods. Luo et al. [20] observed that thick, greasy tongue fur was the most typical clinical symptom of the febrile disease which belonged to the dampness-heat syndrome. We found that the model group's tongue colour was deeper red, while the normal group was pink. The model group rats manifested low spirit and loose stools.

In our study, the body weight of the normal group showed steady growth. We observed different trends of weight gain in the model group and three treatment groups. Shum et al. successfully designed a rat BPH model which was used to explore the effect of dihydrotestosterone in prostate growth in rats [16]. The prostate was a sex hormone dependent organ. We induced prostate hyperplasia in the castrated rats by injecting testosterone propionate. During the administration period after molding, there was no secretory source of sex hormones. As a result, the prostate size and prostate index (PI) of the model group were smaller than the normal group. Compared with the model group, treatment with KLC (7.2 g/kg) for 28 days significantly inhibited the growth of the prostate, which was observed by the prostate size and PI after establishing the BPH model.

Pathological sections of the kidney confirmed necrosis and local oedema in the model group. The prostate sections of the model group showed prostate proliferation. In contrast, the pathological structures of the kidney in the normal and three treatment groups were unaffected. These findings confirmed that the testosterone-induced BPH rat model was successfully established. In addition, administration of KLC (7.2 g/kg) obviously alleviated prostatic hyperplasia, as it was evidenced by thinner epithelium than the model group.

Soy isoflavones from the KLC prescription could replace antiandrogen. Pumpkin seed oil could inhibit testosterone-induced hyperplasia of the prostate [21, 22]. Modern pharmacological studies showed that Huangqi (Astragali Radix), Guizhi (Cinnamomi Ramulus), soy isoflavone, and pumpkin seed oil all have anti-inflammatory and antioxidant activities [23]. COX-2 was a proinflammatory enzyme, which was found to work in concert with many pathophysiological activities and inflammatory diseases [24, 25]. The experimental results showed that the expression of COX-2 increased in the KLC (7.2 g/kg) group, and there were no differences in the other groups compared with the model group. We speculated that the inflammatory factors produced by castration affected the expression level of COX-2 in the prostate tissue of the rat models.

Clinical studies confirmed that the antioxidant defence system decreased in elderly patients with BPH [26, 27]. Other studies also revealed that oxidative stress-mediated pathway was involved in several male urologic disorders [28]. Nrf2 could regulate gene expression through the electrophile response element in response to various forms of oxidative stress [29]. The results indicated that the expression of Nrf2 and NQO1 increased significantly after KLC (3.6 g/kg) treatment compared with the model group. It was suggested that KLC (3.6 g/kg) could activate the antioxidant factors. However, when the dose was 7.2 g/kg, the expression of antioxidant factors decreased and inhibited the proliferation of the prostate.

## 5. Conclusion

In summary, we demonstrated that KLC could effectively inhibit the growth of the prostate and enhance antioxidant activity via increasing the protein expressions of Nrf2 and NQO1. The mechanism of anti-inflammation needed to be further clarified. Our results provided insights into the antioxidant mechanisms of KLC, which was basis for the further research.
